# Complementary Thoracic Endovascular Aortic Repair (TEVAR) after Frozen Elephant Trunk for Residual Type A Aortic Dissection: Perioperative and Mid-Term Outcomes

**DOI:** 10.3390/jcm13103007

**Published:** 2024-05-20

**Authors:** Spyridon N. Mylonas, Ravan Mammadov, Bernhard Dorweiler

**Affiliations:** Department of Vascular and Endovascular Surgery, Faculty of Medicine, University Hospital of Cologne, University of Cologne, 50937 Cologne, Germany; ravan.mammadov@uk-koeln.de (R.M.); bernhard.dorweiler@uk-koeln.de (B.D.)

**Keywords:** frozen elephant trunk, aortic dissection, thoracic endovascular aortic repair, reintervention

## Abstract

**Objectives:** The aim of this retrospective study was to evaluate the results of complementary TEVAR following the frozen elephant trunk (FET) procedure for patients with residual type A aortic dissection (rTAAD) in terms of technical feasibility, safety and mid-term outcomes. **Methods:** This was a retrospective single-centre analysis of patients who received TEVAR after FET for rTAAD from January 2012 up to December 2021. The primary endpoint was technical success. Safety parameters included 30-day/in-hospital morbidity and mortality. Furthermore, mid-term clinical and morphological outcomes were evaluated. **Results:** Among 587 TEVAR procedures, 60 patients (11 with connective tissue disorders) who received TEVAR after FET for rTAAD were identified. The median interval between FET and TEVAR was 28.5 months. Indications for TEVAR after FET were true lumen collapse distal to FET prosthesis (n = 7), dSINE (n = 2), planned completion (n = 13) and aortic diameter progression (n = 38). In forty-seven patients, TEVAR was performed in an elective setting; eight and six patients were operated on in an urgent or emergency setting, respectively. All TEVAR procedures were successfully completed. The 30-day mortality and spinal cord ischemia rates were 1.7%. During a median follow-up of 37 months, two further patients died. Nine patients had to undergo a further aortic intervention: fenestrated stent-graft (n = 3) or open repair of the infrarenal abdominal aorta (n = 6). **Conclusions:** Complementary TEVAR following FET for rTAAD showed excellent technical success and low perioperative risk, supporting the feasibility and safety of this strategy. Despite the favourable mid-term survival, certain patients might require a further aortic procedure.

## 1. Introduction

Acute type A aortic dissection is a potential life-threatening condition that warrants urgent surgical intervention. Currently, a tear-oriented strategy with exclusion or resection of the primary entry tear in the ascending aorta and arch with an open distal anastomosis is recommended to improve survival and increase false-lumen thrombosis [1,2,3]. The extent of surgical strategies varies from the implantation of a supracoronary interposition graft to extensive resection of the ascending aorta and the arch and total arch repair with the frozen elephant trunk (FET) technique. The latter, which is a modification of the conventional elephant trunk technique first suggested by Kato et al. [4], includes a hybrid prosthesis, combining a surgical graft for the replacement of the proximal arch with a distal stent-graft sutured to the distal end of the Dacron graft and deployed antegrade into the descending aorta.

Despite the decline in the previously extremely high postoperative mortality and morbidity for patients undergoing FET, it is estimated that 20% to 40% of patients surviving an acute aortic dissection will develop aortic related complications that may require further treatment [5,6,7,8,9,10,11,12]. Moreover, the incidence of distal stent-graft-induced entry tear (dSINE) is not negligible after FET [13,14,15].

Previously, open surgical repair of the descending aorta after FET with anastomosis to the stent-graft portion used to be applied; however, it had considerable mortality and morbidity rates [10,14,16,17]. Therefore, the concept of an endovascular distal extension through thoracic endovascular aortic repair (TEVAR), which was first suggested by Uchida et al. in 2013, has been adopted in several centres worldwide [16,18,19,20,21,22,23,24,25]. The aim of this retrospective study was to evaluate the results of complementary TEVAR following the FET procedure in patients with residual type A aortic dissection (rTAAD) in terms of its technical feasibility, safety and mid-term outcomes.

## 2. Materials and Methods

### 2.1. Study Design

This was a retrospective single-centre analysis of patients who received TEVAR after FET procedure for residual type A aortic dissection from January 2012 up to December 2021. Our Institutional Ethics Committee approved the data collection (protocol no. 19-1017).

### 2.2. Data Extraction

Data were collected retrospectively using our centre’s prospectively maintained dedicated database. Demographic and baseline characteristics, previous aortic procedures, intraoperative details, clinical outcomes and follow-up data were evaluated.

### 2.3. TEVAR Procedure and Follow-Up Protocol

TEVAR was either the second stage of a planned hybrid procedure or a secondary extension due to disease progression or FET failure. Staged procedures were previously planned in the context of a regularly held multidisciplinary aortic board attended by staff of cardiac and vascular surgery departments. TEVAR procedures were performed in a hybrid operating theatre under general anaesthesia though surgical cut-down of the femoral artery. TEVAR sizing was based only on the proximal landing zone (PLZ) (i.e., the stent-graft portion of the FET), aiming for a maximum of 10% oversize. The intended device overlap was at least 5 cm. TEVAR was usually extended distally to the level of the thoraco-abdominal transition in close proximity to coeliac trunk offspring. Cerebrospinal fluid drainage (CSFD) for spinal cord protection was selectively used, based on the setting of the procedure (elective vs. urgent) and the intended total aortic coverage. All patients were managed according to the standardised postoperative protocol at our intensive care unit (ICU), which entails invasive blood pressure measurement (target mean arterial pressure >80 mmHg), continuous cerebrospinal fluid drainage for at least 48 h and haemoglobin levels of >8 mg/dL.

### 2.4. Follow-Up Protocol and Imaging Data Analysis

Quality control was routinely performed before discharge by means of computed tomography angiography (CTA). The follow-up protocol included visits 6 and 12 months after TEVAR and annually thereafter. Clinical evaluation and morphological analysis were performed through CTA according to a standardised aortic protocol, with electrocardiogram-triggered CTA of the entire aorta at 1 mm slice thickness. The maximum diameter of the descending aorta was evaluated for this analysis. The scans were reviewed independently by two experienced physicians.

### 2.5. Study Endpoints

The primary endpoint was the technical success of complementary TEVAR after FET procedure, defined as the successful implantation of the thoracic stent-graft in the intended location without endoleak type I/III. Safety parameters included 30-day/in-hospital morbidity and mortality. Furthermore, mid-term clinical and morphological outcomes were evaluated.

### 2.6. Statistical Analysis

Descriptive statistics were applied to describe the study population’s characteristics. Continuous data were documented as medians (interquartile ranges) or means (standard deviations) after control of distribution normality. Categorical variables were described as numbers (percentages). The Kaplan–Meier method was applied to analyse late survival. All statistical analyses were conducted in SPSS Statistics, version 28 (Armonk: New York, NY, USA; IBM: New York, NY, USA).

## 3. Results

Between January 2012 and December 2021, 587 TEVAR procedures were performed. Among these, 60 patients (63.3% males) who received TEVAR after previous FET for residual type A aortic dissection were identified and represent this study’s cohort. Median age at time of TEVAR was 66.5 (28–81) years. Eleven patients (18.3%) were diagnosed with connective tissue disorders. The patients’ demographic characteristics and comorbidities are listed in Table 1.

All patients received commercially available FET prostheses: the E-vita Open Hybrid prosthesis (Jotec, Hechingen, Germany) in 47 patients and the Thoraflex prosthesis (Vascutek Ltd./Terumo, Renfrewshire, UK) in 13 patients. The median interval between FET implantation and second-stage TEVAR was 28.5 (1–491) months. Indications for TEVAR after FET were true lumen collapse distal to FET prothesis (n = 7), dSINE (n = 2), planned completion (n = 13) and aortic diameter progression (n = 38). The median aortic diameter at the time of TEVAR was 64 (47–127) mm. In 47 (78.3%) patients, TEVAR was performed in an elective setting, whereas seven (11.6%) and six (1%) patients were operated on in an urgent or emergency setting, respectively. CSF drainage was placed in 25 (41.7%) of the patients. Intraoperative details are described in Table 2.

**Figure 1:**
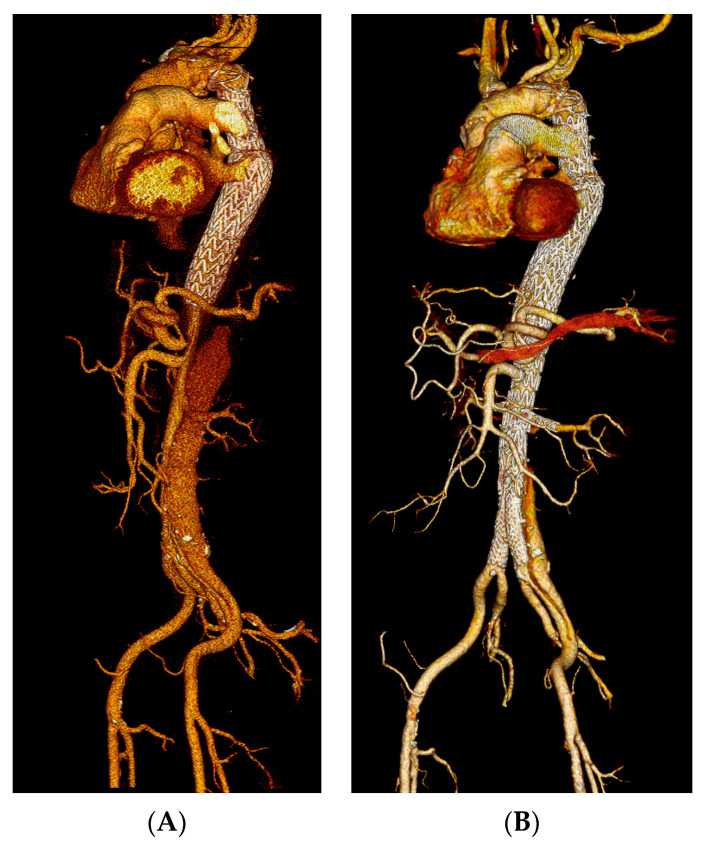
Postoperative CTA of a 67-year-old patient who received TEVAR after a FET procedure due to aortic diameter progression (**A**) followed by a distal completion with a fenestrated stent-graft 8 months later. (**B**) Note the entry tear in LEIA, which was left uncovered to reduce the risk of spinal cord ischemia.

All TEVAR procedures were successfully completed, yielding a technical success rate of 100%. The median ICU stay was 2 days (range, 0–17 days), and the median hospital stay was 7 days (range, 5–31 days). A 73-year-old female patient with a rupture of the false lumen after FET underwent an emergency TEVAR procedure but succumbed on the second postoperative day (POD) due to multi-organ failure (MOF), resulting in a perioperative mortality of 1.7%. None of the electively treated patients died during the first 30 days. Moreover, a further patient who was operated on in an urgent setting developed paraparesis due to SCI, which remained constant despite the applied measures and was discharged to a dedicated rehabilitation centre. Access site complications were observed in four patients, with one of these requiring surgical revision due to haemorrhage. No cerebrovascular events, MI or renal function impairments were recorded within 30 days of TEVAR (Figure 1).

**Figure 2:**
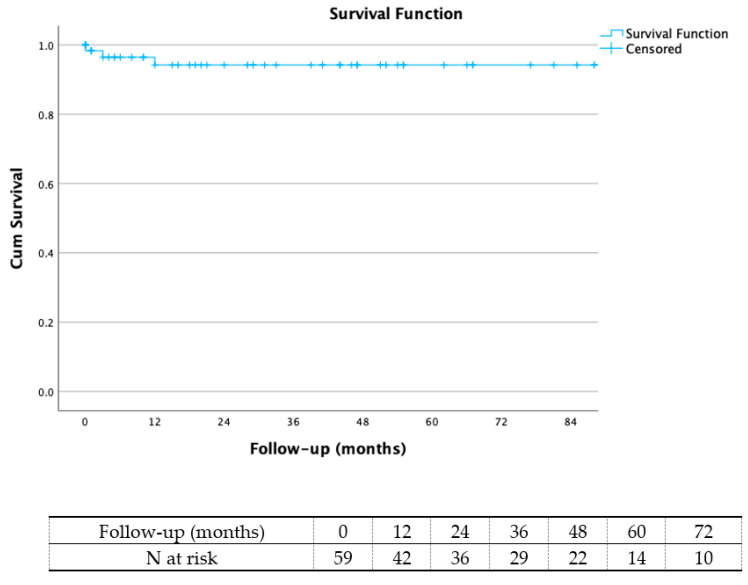
Kaplan–Meier curve of overall survival.

During the median follow-up period of 37 (1–140) months, two further patients (3.3%) died. The above-mentioned patient with permanent paraplegia succumbed due to respiratory complications 4 months after TEVAR, whereas a further patient died 18 months postoperatively due to intracerebral haemorrhage. According to Kaplan–Meier analysis, the overall survival was 94.2% at 60 months (Figure 2).

Nine patients (15%) had to undergo a further aortic intervention due to downstream aortic dilatation during follow-up. These included a distal completion with a fenestrated stent-graft (n = 3) or open repair of the infrarenal abdominal aorta (n = 6). The median aortic diameter at the time of the last follow-up was 52 (25–122) mm.

## 4. Discussion

In our study, TEVAR after FET procedure for residual type A aortic dissection was found to be associated with excellent technical success, low perioperative risk and favourable mid-term survival. However, there was a considerable requirement for further aortic repair during mid-term follow-up.

FET was initially introduced as a ‘standalone’ procedure for the treatment of ascending aorta and/or aortic arch pathologies [4]. During the technique’s evolution, there was a trend among cardiovascular surgeons towards a more proximal surgical aortic arch anastomosis (zone 1 or 2 rather than zone 3), intended to simplify the surgical procedure, and towards the use of shorter stent-graft lengths in order to minimise the risk of spinal cord ischemia [21,26]. However, there is increasing evidence that these modifications have led to a higher need for aortic reinterventions [21,27,28,29,30]. Especially in patients who receive a FET for type A aortic dissection, negative aortic remodelling (either partially thrombotic or patent FL) in the downstream aorta is described to have a prevalence of 27% to 35% and is associated with a need for reintervention in 9.6% to 33% of cases [10,11,31,32].

TEVAR emerged roughly twenty years ago, representing a notable stride in endovascular technology and providing a solution to treat the downstream aorta in cases of both aneurysmal and dissection aortic pathologies. However, major concerns of TEVAR are the lack of an adequate and durable proximal landing zone and the risk of retrograde dissection. FET prostheses can offer a safe docking zone for TEVAR while eliminating the risk of retrograde dissection [33] (Table 3). This is of paramount importance for patients with residual type A aortic dissection, who are usually younger or suffer from connective tissue diseases (18% in our study) and in whom the use of stent-grafts is controversial in the absence of an artificial landing zone [1]. Kozlov et al. recently demonstrated the superiority of FET with additional TEVAR compared to the standard FET procedure in terms of freedom from aortic remodelling and distal reintervention rates [34].

Although secondary aortic reinterventions may be planned or even expected during the initial FET procedure (e.g., initial diameter or presence of multiple re-entry tears in the descending aorta,) there are several adverse events associated with FET requiring unplanned reinterventions (e.g., endoleak, true lumen collapse, dSINE) [5,6,7,8,9,10,11,12,13,14,15]. In our study cohort, a reintervention after FET was planned in 21.7% of the patients, expected in 63.3% and unplanned/unexpected in 15%. This explains the wide interval between FET implantation and TEVAR, ranging from 1 to 491 months, as well as the fact that the vast majority of these reinterventions were conducted in an elective setting. However, 14 patients were operated on in an urgent or emergency setting due to eminent or free rupture of the descending aorta; one of them succumbed on POD 2. This underlines the need for a strict follow-up protocol and proper multidisciplinary decision making for the timing of TEVAR extension.

Another factor that should be considered when applying complementary TEVAR after FET is the risk of SCI. Staging of repair is a proven strategy for minimising the catastrophic risk of SCI, supporting the collateral network concept, whereas the length of aortic coverage is also a known risk factor [35]. We selectively used prophylactic CSF drainage (based on the length of intended aortic coverage and the setting of the procedure) and recorded one case of permanent SCI after urgent TEVAR, which is in line with the findings of other studies [16,18,19,20,21].

A further finding of our study that merits consideration is that 15% of the patients underwent a further aortic intervention due to downstream aortic dilatation during follow-up. In a recent study by Kreibich et al., 15 patients (23%) required additional aortic interventions within 12 months of TEVAR [21]. Other authors have suggested the enhancement of TEVAR with candy-plug embolisation of the false lumen or application of the PETTICOAT or STABILISE techniques to achieve a favourable remodelling of the entire aorta in the midterm [16,18,24]. We successfully performed a distal completion with a fenestrated stent-graft in three patients and an open repair of the infrarenal abdominal aorta in six further patients. In a recent multi-centre study on distal FBEVAR after prior FET, a high technical success rate (92%) and low 30-day mortality (2.6%) were reported [36].

The study is limited by its retrospective and monocentric nature. Despite the fact that the study period exceeded 10 years, the sample size is small. Moreover, by including patients, who had undergone a FET procedure in other centers, a selection bias cannot be excluded, as patients with severe complications after FET could have been omitted. Thus, the informative value of our data may be limited as further statistical analysis for the identification of potential factors affecting the outcome was not feasible.

## 5. Conclusions

This study’s findings suggest that complementary TEVAR following a FET procedure for residual type A aortic dissection is associated with excellent technical success and low perioperative risk, supporting the feasibility and safety of the strategy. Despite the favourable mid-term survival, certain patients might eventually require a further aortic procedure to repair the distal aorta, highlighting the importance of long-term surveillance for these patients.

## Figures and Tables

**Table 1:** Patients’ demographic characteristics and comorbidities.

Age (median, years)	66.5 (28–81)
Gender (male)	38 (63.3%)
HTN	46 (76.7%)
CAD	14 (23.3%)
Renal function	
• 90 mL/min/1.73 m^2^	13 (21.7%)
• 90–60 mL/min/1.73 m^2^	32 (53.3%)
• 60–30 mL/min/1.73 m^2^	13 (21.7%)
• 30–15 mL/min/1.73 m^2^	2 (3.3%)
• <15 mL/min/1.73 m^2^	0
GFR (median, mL/min/1.73 m^2^)	75 (26–120)
COPD	8 (13.3%)
Nicotine consumption	
• active	11 (18.3%)
• history	8 (13.3%)
• no	41 (68.3%)
Cerebrovascular event (stroke, TIA)	6 (10%)
Diabetes mellitus	4 (6.7%)
Connective tissue disorders	
• no	47 (78.3%)
• Marfan	10 (16.7%)
• other	1/60 (1.7%)
• unknown	2 (3.4%)

HTN: hypertension; CAD: coronary artery disease; COPD: chronic obstructive disease; GFR: glomerular filtration rate.

**Table 2:** Intraoperative details.

Duration of TEVAR Procedure (Median, Min)	69 (27–158)
Stent-graft design	
Gore CTAG	31
Gore CTAG with active control	12
Cook Zenith TX2	11
Cook Zenith alpha	4
Bolton Relay NSB Plus	2
No. of devices (median)	2 (1–3)
Device length (median, mm)	150 (100–220)
Fluoroscopy time (median, min)	13 (3–47)

**Table 3:** Contemporary series on TEVAR following FET.

Author	Centre	N	Interval (Months)	Pathology	In-Hospital Mortality	SCI	Follow-Up (Months)	Survival
Fortin et al., 2024 [24]	Montreal, Canada Paris, France	34 *	1	Residual AD	0	0	23	100%
Kreibich et al., 2022 [21]	Freiburg, Germany	66	7	AD (n = 42) TAAA (n = 19) PAU (n = 5)	0	0	12	93.9%
Loschi et al., 2021 [16]	Milan, Italy	20	18	TAAA (n = 5) AD (n = 15)	1 (5%)	1 (5%)	20	95%
Meisenbacher et al., 2022 [19]	Heidelberg, Germany	20	7.7	TAAA (n = 16) AD (n = 4)	1 (5%)	1 (5%)	58.3	95%
Haensig et al., 2020 [18]	Leipzig, Germany	10 *	4.5	TAAA Crawford I (n = 3) TAAA Crawford II (n = 4) AD (n = 3)	1 (10%)	0	8.5	90%
Pan et al., 2017 [20]	Beijing, China	23	24	dSINE (n = 2) residual tear (n = 21)	0	0	33.6	95.7%
Laranjeira Santos et al., 2017 [22]	Lisbon, Portugal	8	1.2	TAAA (n = 6) AD (n = 2)	1 (12.5%)	0	18.7	87.5%
Uchida et al., 2013 [25]	Hiroshima, Japan	10	24	TAAA (n = 6) AD (n = 4)	0	0	18	100%

AD, aortic dissection; TAAA, thoracoabdominal aortic aneurysm; dSINE, distal stent-induced new entry. * including PETTICOAT/STABLISE technique.

## Data Availability

Data are contained within the manuscript.
